# Supplementary figures and images for: Informal genomic surveillance of regional distribution of Salmonella Typhi genotypes and antimicrobial resistance via returning travellers
Source: PLoS Negl Trop Dis. 2019 Sep 12;13(9):e0007620. doi: 10.1371/journal.pntd.0007620 (PMC6741848; doi:10.1371/journal.pntd.0007620)

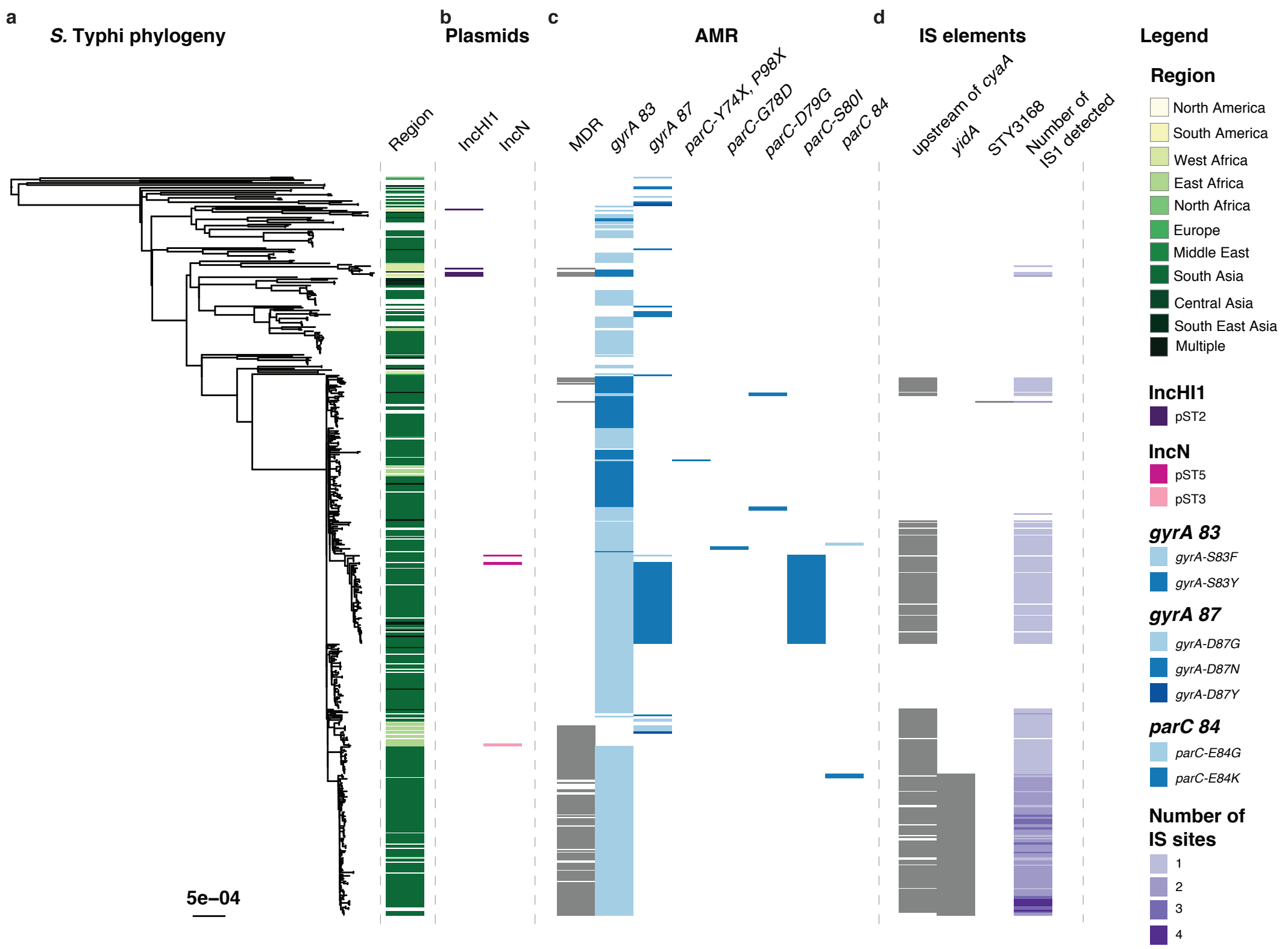

Supplement: S1 Fig — a) The phylogeny of the 533 S. Typhi isolates in the PHE collection is shown on the left with the geographical region of reported travel. b) The presence of IncHI1 or IncN plasmid replicons is shown with the different colours indicating PST. c) The presence of MDR profiles is shown in grey. The presence of point mutations in QRDR genes is shown with the different gradients of blue indicating different mutations. d) Detected IS1 sites of insertion are shown in grey. The total number of IS detected in each of the S. Typhi isolates is shown on the far right. (PDF) [file pntd.0007620.s001.pdf]
